# Effectiveness of HIIT in patients with cancer or cancer survivors: An umbrella and mapping review with meta‐meta‐analysis

**DOI:** 10.1111/sms.14223

**Published:** 2022-08-13

**Authors:** Aida Herranz‐Gómez, Ferran Cuenca‐Martínez, Luis Suso‐Martí, Clovis Varangot‐Reille, Joaquín Calatayud, María Blanco‐Díaz, José Casaña

**Affiliations:** ^1^ Exercise Intervention for Health Research Group (EXINH‐RG), Department of Physiotherapy University of Valencia Valencia Spain; ^2^ Department of Physiotherapy, Faculty of Health Sciences European University of Valencia Valencia Spain; ^3^ Surgery and Medical Surgical Specialities Department, Faculty of Medicine and Health Sciences University of Oviedo Oviedo Asturias Spain

**Keywords:** cancer, cardiorespiratory fitness, high‐intensity interval training, quality of life

## Abstract

**Objective:**

To assess the available evidence on the effectiveness of high‐intensity interval training (HIIT) in addition to first‐choice cancer treatment on cardiorespiratory fitness (CRF), quality of life (QoL), adherence, and adverse effects of HIIT in patients with cancer or cancer survivors.

**Methods:**

An umbrella review and meta‐meta‐analysis (MMA) was performed. A systematic search was conducted in MEDLINE, EMBASE, Cochrane Database, CINAHL, Scopus, SPORTDiscus, and Web of Science until August 2021. Article selection, quality assessment, and risk of bias assessment were performed by two independent reviewers. The MMA were performed with a random‐effects model and the summary statistics were presented in the form of forest plot with a weighted compilation of all standardized mean differences (SMD) and corresponding 95% confidence interval (CI).

**Results:**

Seven systematic reviews were included. Regarding CRF, the addition of HIIT to cancer treatment showed statistically significant differences with a small clinical effect, compared with adding other treatments (SMD = 0.45; 95% CI 0.24 to 0.65). There was no significant difference when compared with adding moderate‐intensity continuous training (MICT) (SMD = 0.23; 95% CI −0.04 to 0.50). QoL showed positive results although with some controversy. Adherence to HIIT intervention was high, ranging from 54% to 100%. Regarding adverse effects, most of the systematic reviews reported none, and in the cases in which they occurred, they were mild.

**Conclusion:**

In conjunction with first‐choice cancer treatment, HIIT has been shown to be an effective intervention in terms of CRF and QoL, as well as having optimal adherence rate. In addition, the implementation of HIIT in patients with cancer or cancer survivors is safe as it showed no or few adverse effects.

## 1 INTRODUCTION

Actually, cancer is one of the leading causes of mortality, with lung cancer being the leading cause of cancer mortality in both sexes (18% of cancer deaths) along with colorectal (9.4%), liver (8.3%), stomach (7.7%), and female breast cancers (6.9%).^1^ Adjuvant and neoadjuvant treatment, such as chemotherapy, radiotherapy, surgery, or hormonal therapy, has increased the survival of patients with cancer,^2^ although they are often accompanied with adverse effects such as cardiotoxicity, fatigue, nausea, mental health problems, disuse, or musculoskeletal disorders.^3^ Therefore, research into safe and effective treatments to mitigate the problems derived from cancer and its treatment is mandatory, which in turn would also contribute to reducing mortality.

Cancer survivors have a significant risk of death from cardiovascular diseases, specifically some types of cancer have a higher than average risk percentage (11.3%): cancer of the larynx (17.3%), prostate (16.6%), uterine body (15.6%), colorectal (13.7%), and breast (11.7%).^4^ One of the main problems in patients with cancer or cancer survivors is physical deconditioning, with several patients showing decreased cardiorespiratory fitness (CRF).^5^, ^6^ In addition to the adverse effects of cancer treatments, other modifiable factors such as the usual sedentary lifestyles of these patients^7^ and aging, influence this variable. Scientific evidence has shown a negative association between CRF level with mortality in cancer survivors.^8^, ^9^ To counteract these issues, exercise is presented as a useful treatment in patients undergoing cancer treatment, thus having a positive impact on the survival rate.^10^ For instance, resistance exercises alone or in combination with aerobic exercise increased muscle mass compared with placebo or non‐treatment control in patients with cancer.^11^ Exercise has been shown to be a safe and effective intervention to improve CRF, strength, fatigue, anxiety, depressive symptoms, or QoL both during and after cancer treatment.^12^, ^13^, ^14^ Exercise prescription is usually based on the FITT principles (frequency, intensity, time, and type of exercise), with intensity being one of the most important parameters to manage in exercise interventions.^15^, ^16^ Including exercise during cancer treatment showed better results in terms of CRF, strength and fatigue when using high intensity compared to low‐moderate intensity.^17^ High‐intensity interval training (HIIT) is a type of exercise that involves intervals of high‐intensity exertion, reaching a percentage of maximal oxygen consumption (VO_2max_) ≥90%, or ≥80% for clinical populations, interspersed with intervals of passive or active recovery at low intensity.^18^, ^19^ This type of training has already been shown to be safe in patients, for example, with cardiac pathology.^20^ Moreover, despite the sedentary behavior of patients with cancer, HIIT does not seem to contribute negatively to the dropout rate in asymptomatic sedentary participants^21^ nor breast patients with cancer.^22^ Therefore, the implementation and adaptation of HIIT may be suitable additional therapeutic option in patients with cancer or cancer survivors.

Thus, the main aim of this umbrella review and meta‐meta‐analysis was to synthesize and analyze the scientific evidence regarding the effectiveness of high‐intensity interval training on cardiorespiratory fitness and QoL as well as its influence on exercise adherence and high‐intensity interval training related adverse effects in patients with cancer or cancer survivors.

## 2 METHODS

We conducted a systematic review of reviews in accordance with the Preferred Reporting Items for Overviews of Systematic Reviews including harm checklist (PRIO‐harms), which consist of 27 items and 56 sub‐items, followed by a 5‐stage process flow diagram.^23^ The protocol of this study was registered in an international register prior to starting the review (PROSPERO, CRD42021275385).

### 2.1 Review inclusion criteria

The selection criteria for this study were based on methodological and clinical factors such as population, intervention, comparison, outcomes, and study design criteria.^24^

#### 2.1.1 Population

The participants selected for the studies were patient older than 18 years with a diagnosis of cancer or cancer survivors, including any type and stage of cancer. The patient´ gender was irrelevant.

#### 2.1.2 Intervention and control

Patients received the first‐choice neo‐ or adjuvant treatment (chemotherapy, radiotherapy, hormone therapy, and surgery). The intervention group received the first‐choice treatment plus HIIT, performed before, during or after cancer‐related treatment. When systematic reviews included interventions other than HIIT, they were included only when the effect of HIIT could be isolated. The comparison group also received the first‐choice treatment, alone or in combination with continuous training, or other treatments (OT) different from HIIT.

#### 2.1.3 Outcomes

The measures used to assess the results and effects were CRF, QoL, adherence, and/or adverse events related to HIIT intervention. We included post‐treatment measurements.

#### 2.1.4 Study design

Systematic reviews (with or without a meta‐analysis) of randomized controlled trials (RCTs) or controlled clinical trials (CCTs) were selected. There were no restrictions for any specific language, as recommended by the international criteria.^25^

### 2.2 Search strategy

We conducted the search for published scientific articles between 1950 and August 24, 2021, in the following databases: MEDLINE (PubMed), EMBASE, Cochrane Database of Systematic Reviews, CINAHL, Scopus, SPORTDiscus, and Web of Science. An additional manual search was realized in Google Scholar. The reference sections of the included studies and original studies were screened manually, and the authors were contacted for further information if necessary. The search strategy combined Medical Subjects' Headings (MeSH [“High‐intensity interval training”]), and non‐MeSH terms (“cancer,” “malignant neoplasm,” “malign neoplasm,” “malignant tumor,” “malign tumor,” “oncology,” “high‐intensity interval exercise,” “high‐intensity intermittent training,” and “high‐intensity intermittent exercise”) adding a Boolean operator (AND and/or OR) to combine them. Appendix 1 shows the search strategy, which was adapted for each database.

Two independent reviewers (A.H.G and C.V.R) conducted the search using the same methodology, and differences during this phase were resolved by consensus. Rayyan QCRI software was employed to remove duplicates and hand‐checked and to perform the screening process.^26^

### 2.3 Selection criteria and data extraction

First, the two independent reviewers (A.H.G and C.V.R) conducted a data analysis assessing the relevance of the reviews regarding the study questions and objectives. This initial analysis was performed based on information from each study's title, abstract, and keywords. If there was no consensus or if the abstracts contained insufficient information, the full text was reviewed. The second phase of the analysis using the full text was performed to assess whether the studies met all the inclusion criteria. Differences between the reviewers were resolved by discussion and consensus moderated by a third reviewer (J.C.G).^27^ The data described in the results were extracted by means of a structured protocol that ensured that the most relevant information was obtained from each study.^28^

### 2.4 Methodological quality assessment

The methodological quality of the included systematic reviews was assessed by two independent reviewers (A.H.G and C.V.R) based on the Modified Quality Assessment Scale for Systematic reviews (AMSTAR), developed by Barton et al., which was found to be a valid and reliable tool for assessing the methodological quality of systematic reviews. The scale has a total of 13 items, each one rated between 0 and 2 (“yes” scoring 2; “in part· scoring 1; “no” scoring 0), and the maximum possible score is 26 points, with a score of 20 or more points being considered high quality.^29^

Disagreements on the final quality assessment score between the reviewers were resolved by consensus with a third reviewer (J.C.G). The inter‐rater reliability was calculated using the kappa coefficient (*κ*): (1) *κ* > 0.7 indicates a high level of agreement between the reviewers; (2) *κ* of 0.5–0.7 indicates a moderate level of agreement; and (3) *κ* < 0.5 indicates a low level of agreement.^30^

### 2.5 Risk of bias assessment

The two independent reviewers (A.H.G and C.V.R) assessed the risk of bias in the selected reviews with the Risk of Bias in Systematic Reviews tool (ROBIS), which evaluates the quality across 4 domains: (1) study eligibility criteria; (2) study identification and selection; (3) data collection and study appraisal; and (4) synthesis and findings. The ROBIS tool includes signaling questions to evaluate specific domains and the overall risk of bias is therefore provided as low, high, or unclear.^31^

Disagreements between the reviewers were resolved through consensus and mediation by a third reviewer (J.C.G). The inter‐rater reliability was estimated employing the same κ cut‐offs described in methodological quality assessment.

### 2.6 Overall strength of the evidence

We assessed the strength of the evidence across the systematic reviews using the Physical Activity Guidelines Advisory Committee (PAGAC). For the PAGAC analysis, the findings were evaluated according to five criteria: (1) applicability of the study sample, exposures, and outcomes to the research question; (2) generalizability to the population of interest; (3) risk of bias or study limitations; (4) quantity and consistency of findings across studies; and (5) magnitude and precision of the effect. The strength of the evidence was classified according to the PAGAC as strong, moderate, limited, or not assignable.^32^

### 2.7 Evidence map

The scientific evidence from each meta‐analysis was presented in a mapping using the following criteria:

1. Number of studies (figure size): The size of each figure is directly proportional to the number of original studies included in each of the meta‐analysis.
2. Type of comparator (bubble color): The type of comparison intervention determines each figure's color. The risk of bias of the study was represented by the color of the outline of the figure. The score for methodological quality on the AMSTAR scale, out of 26 points, was indicated within the bubble.
3. Effect size (*x*‐axis): Each of the reviews was classified according to the size effect as described by Hopkins.^33^ The categorization of the effect size is described in the *Data synthesis and analysis* section.
4. Strength of findings (*y*‐axis): The reviews were sorted into the following 4 categories according to the Physical Activity Guidelines Advisory Committee (PAGAC): strong, moderate, limited, or not assignable.

### 2.8 Data synthesis and analysis

#### 2.8.1 Meta‐Analysis of pooled results

Meta‐Analyses of pooled results were performed using *Meta XL*, version 5.3 (EpiGear International, Queensland, Australia).^34^ We used the same inclusion criteria for the systematic review and meta‐analysis but added 2 criteria: (1) The Results section contained detailed information on the comparative statistical data (mean, standard deviation, and/or 95% confidence interval [CI]) of the main variables, and (2) data for the analyzed variables were represented in at least three meta‐analyses. We presented the summary statistics in the form of forest plots,^35^ which consist of a weighted compilation of all standardized mean differences (SMDs) and corresponding 95% CI reported by each study and provide an indication of heterogeneity among the studies. To obtain a pooled estimate of the effect in the meta‐analysis of the heterogeneous studies, we performed a random‐effects model, as described by DerSimonian and Laird.^36^ The estimated SMDs were interpreted as described by Hopkins et al. an SMD of 4.0 was considered to represent an extremely large clinical effect; 2.0–4.0 a very large effect; 1.2–2.0 a large effect; 0.6–1.2 a moderate effect; 0.2–0.6 a small effect; and 0.0–0.2 a trivial effect.^33^

When the results from meta‐analyses were reported as mean difference (MD) or weighted mean difference (WMD), there were re‐expressed as SMD. To realize it, we entered in the primary studies in order to re‐run the meta‐analyses using *Meta XL*, version 5.3 (EpiGear International, Queensland, Australia).^34^ If necessary, CI and standard error (SE) where converted in standard deviation (SD) using the formulas recommended by the Cochrane Handbook for Systematic Reviews of Interventions version 6.2: *SD* = √(N)*(upper limit–lower limit)/3.92 and *SD* = √(N)*SE, respectively.^37^ If the authors provided only graphics, we extracted data using the software *WebPlotDigitizer* version 4.5 (Pacifica, California, USA).^38^, ^39^, ^40^

#### 2.8.2 Analysis of the influence of duplicity of primary studies

To evaluate the robustness of our meta‐analyses of pooled results, we run an equivalent quantitative analysis where primary studies appear only once. The purpose of this analysis is to assess how the results of studies included in multiple meta‐analyses might affect the final results.

#### 2.8.3 Analysis of the heterogeneity

We estimated the degree of heterogeneity among the studies by employing Cochran's *Q* statistic test (*p* < 0.1 was considered significant) and the inconsistency index (*I* ^2^).^41^ An *I* ^2^ > 25% is considered to represent low heterogeneity, while an *I* ^2^ > 50% is considered medium, and an *I* ^2^ > 75% is considered to represent large heterogeneity.^42^ The *I* ^2^ index is complementary to the *Q* test, although it has a similar problem with power as does the *Q* test with a small number of studies.^42^ A study was therefore considered heterogeneous when it fulfilled one or both of the following conditions: (1) the *Q*‐test was significant (*p* < 0.1), and (2) the result of *I* ^2^ was >75%.

#### 2.8.4 Detection of publication bias

To detect publication bias, we performed a visual evaluation of the DOI plot,^43^ seeking asymmetry. In addition, quantitative measure of Luis Furuya‐Kanamori (LFK) index was performed. This index has been shown to be more sensitive than the Egger test to detect publication bias in meta‐analysis of a low number of studies.^44^ LFK index within ±1 represents no asymmetry; LFK index exceeds ±1 but within ±2 represents minor asymmetry and LFK index exceeds ±2 involve major asymmetry.^44^

## 3 RESULTS

The study screening strategy is presented in the form of a flow chart (Appendix 2). Seven systematic reviews met the inclusion criteria, six of the included studies were systematic reviews and meta‐analysis,^45^, ^46^, ^47^, ^48^, ^49^, ^50^ while the remaining study was systematic reviews without quantitative synthesis.^51^ The characteristics of the included studies (study design, original studies included, demographic characteristics, interventions, outcomes, and results) are presented in Tables 1 and 2. Some of the original studies were included in several reviews, with a duplication rate of 44%, but none of the included reviews presented exactly the same studies (Appendix 3).

**TABLE 1:** Description of systematic reviews included

Study	N°, design of studies, sample	Cancer type and patient's characteristics	Time point of the treatment	Outcomes		
			Intervention and control group	N° of studies includes in meta‐analysis	Scales of measurement	Results
Palma et al., 2021	7 RCTs and 1 CCT *N* = 896	Non‐small cell lung carcinoma, breast cancer, bladder cancer, colorectal liver metastasis, non‐metastatic rectal cancer, unspecific non‐metastatic cancer Mean age range: 47–73 years First‐choice treatment: CT, RT or surgery	Prehabilitation Intervention: HIIT Control: OT	Cardiovascular Fitness		
				6 RCTs and 1 CCT	VO_2max_	HIIT training showed statistically significant improvements of VO_2max_ (MD = 2.76; 95% CI 1.65 to 3.86) when compared with OT.
				Quality of Life		
				N/A	EORTC‐QLQ C30, SF‐36 and FACT‐L	HIIT training seemed to be effective to improve quality of life.
				Adherence and Adverse events		
				N/A	N/A	Adherence ranged from 71 to 96%. Five studies reported no adverse events, and one reported few adverse events (1/134 seizure episode and 5/134 leucopenia and/or blood pressure increase).
Smyth et al., 2021	8 RCTs *N* = 384	Colorectal, liver metastasis, non‐small cell lung carcinoma, bladder cancer Mean age range: 61 to 72 years First‐choice treatment: surgery	Prehabilitation Intervention: HIIT Control: OT or MICT	Cardiovascular Fitness		
				5 RCTs	VO_2max_	HIIT training showed non‐statistically significant effect on VO_2max_ (MD = 0.83; 95% CI −0.51 to 2.17) against OT and MICT.
				Adherence and Adverse events		
				N/A	N/A	Adherence ranged from 54% to 95%. Six did not report any adverse events and one reports mild adverse events (discomfort with the cycle ergometer and mild leg pain post‐exercise).
Tsuji et al., 2021	12 RCTs *N* = 639	Breast cancer Mean age range: 31 to 68 years First‐choice treatment: CT	Rehabilitation Intervention: HIIT Control: OT or MICT	Cardiovascular Fitness		
				N/A	VO_2max_	HIIT training seemed to be effective to improve cardiorespiratory fitness in cancer survivors. However, during active treatment, there were contrasting results.
				Quality of Life		
				N/A	EORTC‐QLQ C30	HIIT training seemed to be effective to improve quality of life, however, it was evaluated only in one study.
				Adherence and Adverse events		
				N/A	N/A	Adherence ranged from 57 to 97% during active treatment, and 57 to 75% in cancer survivors. All studies that assessed the presence of adverse events found none, either during active treatment or in survivors.
Maginador et al., 2020	1 RCT and 1 CCT *N* = 94	Breast cancer Mean age range: 44 to 49 years First‐choice treatment: CT	Rehabilitation Intervention: HIIT Control: OT	Cardiovascular Fitness		
				1 RCT and 1 CCT	VO_2max_	HIIT training showed a statistically significant large effect size compared with no intervention (*d* = 1.79; 95% CI 0.28 to 3.29).
Wallen et al., 2020	11 RCTs and 1 CCT *N* = 516	Colorectal liver metastasis, colorectal cancer, lung cancer, rectal cancer, bladder cancer, breast cancer, mixed cancer, testicular cancer Mean age range: 44–84 years First‐choice treatment: CT, RT, surgery or hormonal therapy	Prehabilitation and rehabilitation Intervention: HIIT Control: OT or MICT	Cardiovascular Fitness		
				HIIT vs OT: 9 RCTs and 1 CCT HIIT vs MICT: 4 RCTs	VO_2max_	HIIT training showed a statistically significant effect on VO_2max_ (WMD = 2.11, 95% CI 0.75 to 3.47) against OT, but non‐significant against MICT (WMD = 2.04; 95% CI −0.75 to 4.83).
				Adherence and Adverse events		
				N/A	N/A	Adherence ranged from 71 to 100%. Eleven studies did not report any serious adverse events, one did not specify an adverse or serious adverse event. One did report mild adverse events (post‐exercise hypotension, acute knee pain, nausea and one episode of sciatica‐related symptoms).
Mugele et al., 2019	10 RCTS and 2 CCTs *N* = 448	Non‐small cell lung cancer, colorectal cancer, rectal cancer, testicular cancer, breast cancer, unspecific cancer (cervical, colon, ovarian, vaginal, melanoma, non‐invasive urothelial carcinoma and non‐Hodgkin's lymphoma) Mean age range: 43 to 64 years First‐choice treatment: CT, RT, surgery, hormonal therapy, immunotherapy	Prehabilitation or Rehabilitation Intervention: HIIT Control: OT or MICT	Cardiovascular Fitness		
				HIIT vs OT: 4 RCTs and 1 CCT HIIT vs MICT: 4 RCTs	VO_2max_	HIIT training showed a statistically significant effect on VO_2max_ (MD = 3.73, 95% CI 2.07 to 5.39) against OT, but non‐significant against MICT (MD = 1.36; 95%CI −1.62 to 4.35).
				Quality of Life		
				N/A	EORTC‐QLQ C30 and SF‐36	Two studies reported no statistically significant differences between HIIT and OT in overall health and quality of life, nor in the subscales of pain, fatigue, and insomnia. However, one study reported that HIIT training had statistically significant differences in the mental component, general health, vitality, social functioning.
				Adherence		
				N/A	N/A	Adherence ranged from 83.6 to 100%.
Blackwell et al., 2018	7 RCTs *N* = 303	Breast Cancer, non‐small cell lung cancer, colorectal liver metastasis, lung cancer, colorectal cancer and unspecific cancer Mean age range: 54 to 64 years First‐choice treatment: CT, RT, surgery, hormonal therapy	Prehabilitation or Rehabilitation Intervention: HIIT Control: OTor MICT	Cardiovascular Fitness		
				HIIT vs OT: 4 RCTs HIIT vs MICT: 3 RCTs	VO_2max_	HIIT training shown a statistically significant effect on VO_2max_ (WMD = 2.93; 95% CI 1.66 to 4.19) against OT, but non‐significant against MICT (WMD = 0.04, 95% CI −3.16 to 3.23).
				Quality of Life		
				N/A	EORTC‐QLQ C30 and SF‐36	There were contradictory results according to the efficacy of HIIT training to improve quality of life.

Abbreviations: CCT, non‐randomized controlled trial; CI, Confidence Interval; CI, Chemotherapy; EORTC‐QLQ C30, European Organization for Research and Treatment of Cancer questionnaire; FACT‐L, functional Assessment of Cancer Therapy‐Lung survey; HIIT, high‐intensity interval training; MD, mean difference; MICT, moderate‐intensity continuous training; N/A, not applicable; OT, other treatment; RCT, randomized controlled trial; RT, Radiotherapy; SF‐36, 36‐ item short form survey; VO_2max_, maximal oxygen uptake; WMD, weighted mean difference.

**TABLE 2:** Interventions included in each of the systematic reviews

Study	Group	Exercise modality	Intervention characteristics			Intervention duration	Frequency	Session duration
			Protocol (Distribution and exercise type)	Work interval (Duration and intensity)	Rest interval (Duration and intensity)			
Palma et al., 2021	HIIT	AE‐HIIT, AE‐HIIT+RT or AE‐HIIT+MICT	WU: 5–10 min	WU: 50–60% WR_peak_ or 50 W		2 to 16 weeks	2 to 5 times per week	11 to 40 min
			HIIT: Cycle ergometer 1–2 sets, 3–6 intervals 4–5 min rest between sets Work/rest ratio: 1:1, 2:1, 3:1	HIIT: 15 s‐5 min 75–95% VO_2max_, 80–100% WR_peak_ or “all out” effort, 85–95% HR_max_	HIIT: 15 s‐2 min Passive rest or Active (<60% VO_2max_ or 50 W)			
			RT: 2–3 sets x 5–12 repetitions MICT: 20 min	RT: 70–100% 1RM MICT: Intensity not reported				
			CD: 5 min	CD: 30%WR_peak_ or 50 W				
	Control	OT	OT: Maintain usual physical activity, exercise recommendations					
Smyth et al., 2021	HIIT	AE‐HIIT	WU: 2–5 min	WU: 50% WR_peak_, 50 W or unloaded		3 to 6 weeks	2 to 5 times per week	20 to 45 min
			HIIT: Cycle ergometer 1–2 sets, 2–40 intervals 4 min rest between sets Work/rest ratio: 0.25:1, 0.75:1, 1:1, 1:2, 2:1	HIIT: 15 s‐5 min >60% VO_2max_, 80–100% WR_peak_ or “all out” effort, 100–115% max load (W), 85–90% PPO or 13–15 RPE	HIIT: 5 s‐4 min Passive rest or Active rest (<60% VO_2max_, 30% WR_peak_, 50 W, 80–85% power anaerobic threshold or unloaded)			
			CD: 2–5 min	CD: 30% WR_peak_ or unloaded				
	Control	OT or MICT	OT: Maintain habitual physical activity, exercise recommendations dietary regimen MICT: 20 min, cycle ergometer	MICT: 80–85% power anaerobic threshold				
Tsuji et al., 2021	HIIT	AE‐HIIT or AE‐HIIT+RT	WU: 3–15 min	WU: 50% VO_2max_, 10–50% WR_peak_ or unloaded		6 to 16 weeks	2 to 3 times per week	10 to 40 min
			HIIT: Cycle ergometer or treadmill 1 set, 3–10 intervals Work/rest ratio: 0.25:1, 1:1, 1:2, 3:1	HIIT: 30 s‐3 min 85–100% VO_2max_, 90% WR_peak_ or 90–95% HR_max_	HIIT: 1–3 min Passive rest or Active rest (<60% VO_2max_, 10% WR_peak_, 70% HR_max_, unloaded or light resistance)			
			RT: 2–3 sets x 8–12 repetitions	RT: 60–80% 1RM				
			CD: 3–5 min	CD: 50% VO_2max_, 10–50% WR_peak_ or unloaded				
	Control	OT or MICT	OT: maintain usual physical activity or exercise recommendations MICT: 20 min, cycle ergometer, treadmill or walk	MICT: 55–65% VO_2max_, 50–65% WR_peak_ or 9–13 RPE				
Maginador et al., 2020	HIIT	AE‐HIIT	WU: 10 min	WU: 60–70% HR_max_ or 10% PPO		6 to 8 weeks	3 times per week	30 to 50 min
			HIIT: Cycle ergometer or treadmill 1 set, 4–7 intervals Work/rest ratio: 1:2, 1:0.6					
			CD: 3–5 min	CD: Not reported				
	Control	OT	OT: maintain usual physical activity					
Wallen et al., 2020	HIIT	AE‐HIIT	WU: 5–10 min	WU: 10–50% WR_peak_, 50 W, 50–70% HR_peak_ or unloaded		3 to 12 weeks	2 to 5 times per week	8 to 45 min
			HIIT: Cycle ergometer or treadmill 1–2 sets, 2–40 intervals 4 min rest between sets Work/rest ratio: 0.75:1, 1:0.75, 1:1, 1:2, 2:1, 1:4	HIIT: 15 s‐5 min 65–95% VO_2max_, 80–100% WR_peak_, 70–90% HR_max,_ 85–95% HR_peak_ or 13–17 RPE	HIIT: 15 s‐5 min Passive rest or Active rest (<60% VO_2max_, 10% WR_peak_, 50–70% HR_peak_, 11–13 RPE or light resistance)			
			CD: Nil‐5 min	CD: 30% WR_peak_, 50 W or unloaded				
	Control	OT or MICT	OT: Maintain usual physical activity or exercise recommendations MICT: 15–50 min, cycle ergometer, treadmill or walk	MICT: 55–70% VO_2max_, 50–70% HR_peak_ or 50–60% HR_max_				
Mugele et al., 2019	HIIT	AE‐HIIT	WU: 5–10 min	WU: 50–70% HR_peak_, 50% PPO or 5% ventilatory threshold 50% PPO		21 days to 12 weeks	3 times per week	20 to 40 min
			Cycle ergometer or treadmill 1–2 sets, 2–40 intervals 4 min rest between sets Work/rest ratio: 1:1, 1:0.75, 1:2	HIIT: 15 s‐5 min 75–95% VO_2max_, 85–95% HR_peak_, 80–100% PPO or 13–17 RPE	HIIT: 15 s‐4 min Passive rest or Active rest (50–60% VO_2max_, 50–70% HR_peak_, 11–13 RPE, 5–10% ventilatory threshold or light resistance)			
			CD: Nil‐5 min	WU: 50–70% HRpeak, 30% PPO or 5% ventilatory threshold				
	Control	OT or MICT	OT: maintain usual physical activity or exercise recommendations MICT: 20–75 min, cycle ergometer, treadmill or walk	MICT: 55–70% VO_2max_, 60% HR_max_, 50–70% HR_peak_,				
Blackwell et al., 2018	HIIT	AE‐HIIT	WU: 5–10 min	WU: 10–50% WR_peak_, 50 W, 50–70% HR_peak_ or unloaded		3 to 6 weeks	2–3 times per week	20 to 30 min
			HIIT: Cycle ergometer or treadmill 1–2 sets, 2–40 intervals 4 min rest between sets Work/rest ratio: 1:1, 1:2, 1:0.75	HIIT: 15 s‐4 min 80–95% VO_2max_, 80–100% WR_peak_ or 85–95% HR_peak_	HIIT: 15 s‐4 min Passive or active (<60% VO_2max_, 50–70% HR_peak_, or light resistance) rest			
			CD: Nil‐5 min	CD: 30% WR_peak_, 50 W or unloaded				
	Control	OT or MICT	OT: Maintain habitual physical activity MICT: 15–50 min, cycle ergometer, treadmill or walk	MICT: 55–70% VO_2max_, 50–70% HR_peak_ or 50–60% HR_max_				

Abbreviations: AE‐HIIT, aerobic high‐intensity interval training; CD, cold down; HIIT, high‐intensity interval training; HR_max_, maximal heart rate; HR_peak_, peak heart rate; MICT, moderate‐intensity continuous training; OT, other treatment; 1RM, one‐repetition maximum; PPO, peak power output; RPE, Borg Rating of Perceived Exertion; RT, resistance training; VO_2max_, maximal oxygen uptake; W, Watts; WR_max_, highest work rate; WR_peak_, work rate peak; WU, warm up.

### 3.1 Characteristics of the included systematic reviews

Our umbrella review and meta‐meta‐analysis (MMA) included seven systematic reviews, including 33 original studies, 30 RCTs and three CCTs, with a total of 2501 patients.

Regarding the study population, only two systematic reviews exclusively analyzed patients with breast cancer^47^, ^51^ while the remaining systematic reviews covered different types of cancers such as colorectal, prostate, breast, bladder, or non‐small cell lung carcinoma, among others.

The entire study population was undergoing or awaiting first‐choice cancer treatment, including radiotherapy, chemotherapy, hormonal therapy, immunotherapy, and/or surgery. During this process, patients in the intervention group also performed aerobic HIIT in five articles, and in two of them, they included aerobic HIIT and resistance exercises.^45^, ^51^ The control group added OT or moderate‐intensity continuous training (MICT) to the first‐choice cancer treatment. In terms of the timing of the intervention, two studies conducted prehabilitation,^45^, ^46^ one study conducted rehabilitation during cancer treatment,^47^ one study conducted rehabilitation during and after treatment,^51^ while the remaining three conducted both prehabilitation and intervention during and after treatment.^48^, ^49^, ^50^

### 3.2 Results of the methodological quality

Regarding the methodological quality, the scores ranged from 12 to 18 points out of a possible 26. All the systematic reviews presented low methodological quality with a score of lower than 20 points. The items with the lowest scores were those related to address the level of evidence in the conclusion, exclusion of the studies, and heterogeneity in the meta‐analyses (Table 3). The inter‐rater reliability of the methodological quality assessment was high (*κ* = 0.767).

**TABLE 3:** Quality assessment scores

Study	1	2	3	4	5	6	7	8	9	10	11	12	13	Score
Palma et al., 2021	0	2	0	1	1	2	1	2	2	0	2	1	0	**14**
Smyth et al., 2021	2	2	1	0	1	2	0	2	1	2	2	1	0	**16**
Tsuji et al., 2021	2	2	1	1	1	1	1	1	1	0	0	1	0	**12**
Maginador et al., 2020	2	1	2	2	0	2	0	2	2	0	2	1	0	**16**
Wallen et al., 2020	1	1	1	2	0	1	0	1	1	1	2	1	0	**13**
Mugele et al., 2019	2	1	2	2	1	1	2	0	2	2	2	1	0	**18**
Blackwell et al., 2018	1	2	2	1	2	1	1	2	1	0	2	1	0	**16**

*Note*: 1. Explicitly described to allow replication; 2. Adequate number and range of databases; 3. Alternative searches; 4. Adequate range of key words: “Cancer,” “Neoplasm,” “Oncology,” “HIIT,” “High‐intensity interval training,” “Cardiorespiratory fitness,” “Quality of Life;” 5. Non‐English‐language papers included in the search; 6. Inclusion criteria explicitly described to allow replication; 7. Excludes reviews which do not adequately address inclusion (cancer and HIIT/igh‐intensity interval training) and exclusion (High‐intensity continuous training) criteria; 8. Two independent reviewers assessing selection bias; 9. Quality assessment explicitly described to allow replication; 10. Meta‐analysis conducted on only homogeneous data or limitations to homogeneity discussed; 11. Confidence intervals/effect sizes reported where possible; 12. Conclusions supported by the meta‐analysis or other data analysis findings 13. Conclusions address levels of evidence for each intervention/comparison.

### 3.3 Results of the risk of bias

**FIGURE 1:**
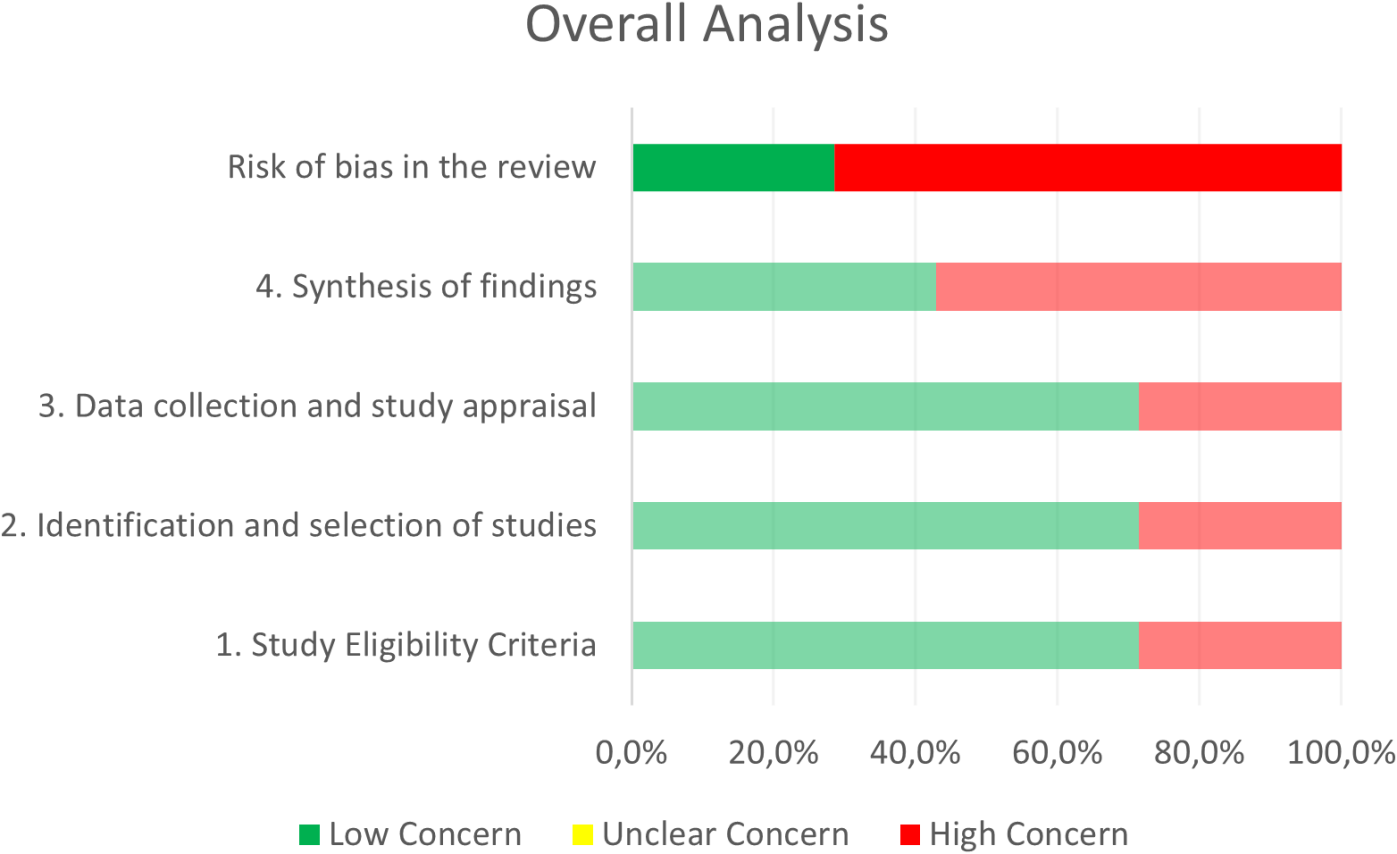
Graphical representation for ROBIS results

Regarding risk of bias, two systematic reviews had a low risk of bias,^47^, ^50^ while the remaining five had a high risk of bias. The domain “synthesis of findings” presented the highest risk of bias (Table 4 and Figure 1). The inter‐rater reliability for the risk of bias assessment was high (*κ* = 0.842).

**TABLE 4:** Risk of bias assessment in systematic reviews through ROBIS scale

Study	Phase 2				Phase 3
	1. Study Eligibility Criteria	2. Identification and selection of studies	3. Data collection and study appraisal	4. Synthesis of findings	Risk of bias in the review
Palma et al., 2021	H	H	L	H	H
Smyth et al., 2021	L	L	L	H	H
Tsuji et al., 2021	H	H	H	H	H
Maginador et al., 2020	L	L	L	L	L
Wallen et al., 2020	L	L	H	L	H
Mugele et al., 2019	L	L	L	H	H
Blackwell et al., 2018	L	L	L	L	L

Abbreviations: H, high concern; L, low concern.

### 3.4 Evidence map

**FIGURE 2:**
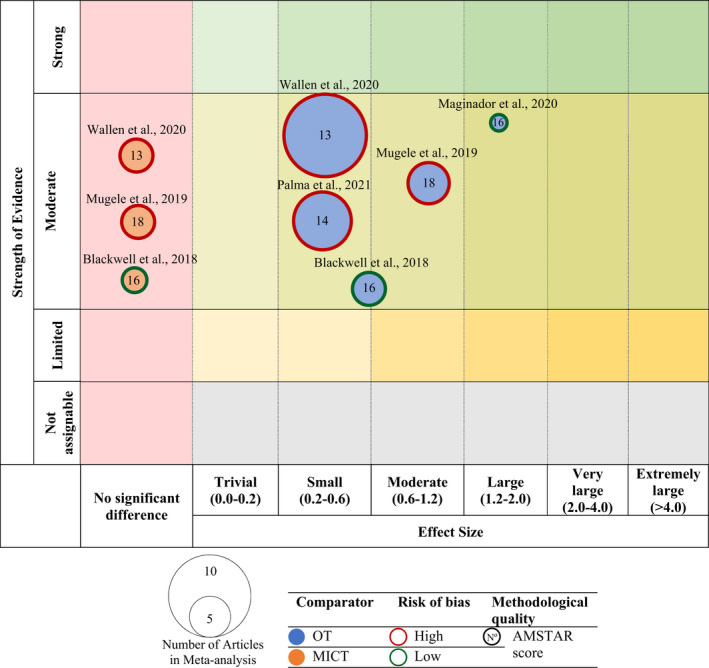
Evidence map of meta‐analyses on cardiorespiratory fitness. Blue bubble: other treatment (OT) as a comparator; Orange bubble: moderate‐intensity continuous training (MICT) as a comparator; Red shape: high risk of bias; Green shape: low risk of bias.

Figure 2 presents the results of the evidence map for the seven studies. Table 5 shows the results of the strength of evidence according to PAGAC.

**TABLE 5:** Summary of findings and quality of evidence according to Physical Activity Guidelines Advisory Committee Grading Criteria (PAGAC)

2018 Physical activity guidelines advisory committee grading criteria						Effect	Evidence
Systematic review research questions (N of studies)	Applicability	Generalizability	Risk of bias or study limitations	Quantity and consistency	Magnitude and precision of effect	Absolute (95% CI)	
VO_2max_, vs other treatment (4)	Strong	Strong	Limited	Limited	Strong	0.45 (0.24; 0.65)	Moderate
VO_2max_, vs MICT (3)	Strong	Strong	Limited	Limited	Strong	0.23 (−0.04; 0.50)	Moderate

Abbreviations: 95% CI, 95% confidence interval; MICT, moderate intensity continuous training; VO_2max_, maximal oxygen uptake.

### 3.5 Cardiorespiratory fitness

Seven studies evaluated CRF when implementing HIIT in patients with cancer or cancer survivors. Five of the studies found a significant increase in VO_2max_ when implementing HIIT versus OT, both added to the first‐choice cancer treatment. Three of these studies used prehabilitation and rehabilitation HIIT,^48^, ^49^, ^50^ one only prehabilitation,^45^ and one rehabilitation.^47^ However, there was no statistically significant improvement when compared with adding MICT during prehabilitation and rehabilitation.^48^, ^49^, ^50^ Smyth et al. found no significant differences when comparing HIIT against OT or MICT plus first‐choice cancer treatment during prehabilitation,^46^ while Tsuji et al. found that CRF improved in cancer survivors, but not in the on‐treatment intervention with a rehabilitation intervention.^51^

**FIGURE 3:**
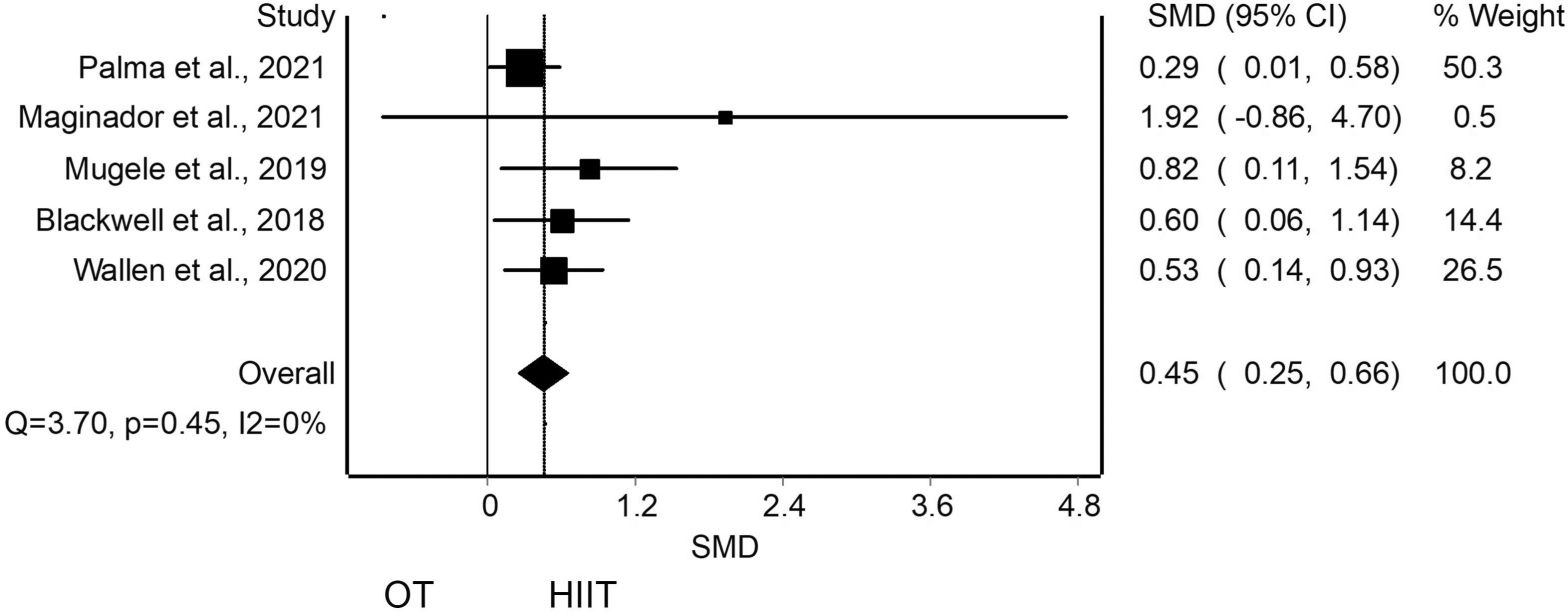
Synthesis forest plot for cardiorespiratory fitness for high‐intensity interval training (HIIT) against other treatment (OT). This forest plot summarizes the results of included studies (standardized mean differences [SMDs], and weight). The small boxes with the squares represent the point estimate of the effect size and sample size. The lines on either side of the box represent a 95% confidence interval (CI)

With regard to the quantitative analysis, the meta‐analysis of pooled results of CRF for adding HIIT against OT did reveal a statistically significant differences with small clinical effect in favor of HIIT in four studies (SMD = 0.45; 95% CI 0.24 to 0.65) with no evidence of significant heterogeneity (*Q* = 2.61, *p* = 0.45, *I* ^2^ = 0%)^45^, ^47^, ^48^, ^49^, ^50^ (Figure 3). The shape of the funnel and DOI plot presented asymmetry, and the LFK index showed major asymmetry (LFK = 4.16), indicating the risk of publication bias (Appendix 4A). The analysis of duplicity reveals almost no influence of the duplicity (SMD = 0.49; 95% CI 0.23 to 0.76; Appendix 5A). The certain of evidence was moderate, showing that HIIT increases VO_2max_ compared with OT (Table 5).

**FIGURE 4:**
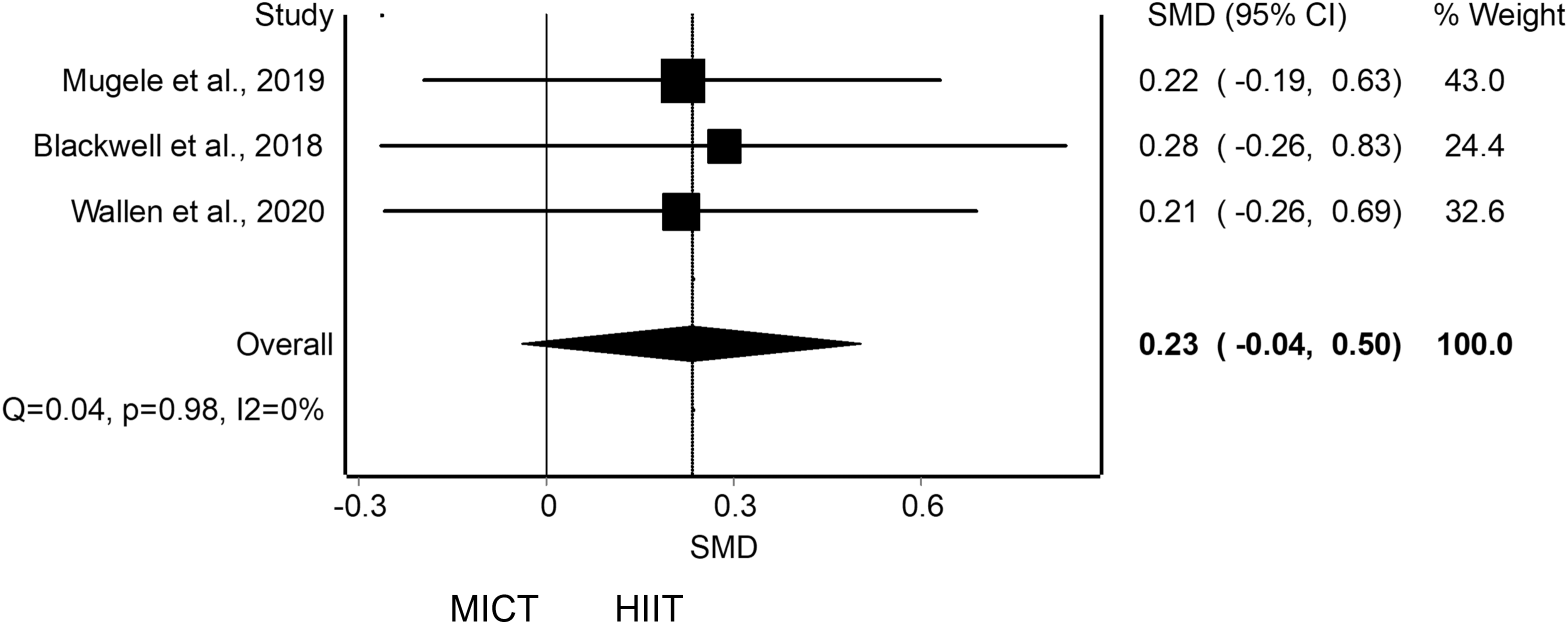
Synthesis forest plot for cardiorespiratory fitness for high‐intensity interval training (HIIT) against moderate‐intensity continuous training (MICT). This forest plot summarizes the results of included studies (standardized mean differences [SMDs], and weight). The small boxes with the squares represent the point estimate of the effect size and sample size. The lines on either side of the box represent a 95% confidence interval (CI).

The meta‐analysis of pooled results of CRF for adding HIIT against MICT did not reveal a statistically significant differences in three studies (SMD = 0.23; 95% CI −0.04 to 0.50) with no evidence of significant heterogeneity (*Q* = 0.04, *p* = 0.98, *I* ^2^ = 0%)^48^, ^49^, ^50^ (Figure 4). The shape of the funnel and DOI plot presented asymmetry, and the LFK index showed minor asymmetry (LFK = 1.79), indicating risk of publication bias (Appendix 4B). The analysis of duplicity reveals an influence on the estimated effect with an overestimation of the effect (SMD = 0.08; 95% CI −0.27 to 0.43; Appendix 5B). The certain of evidence was moderate, showing that HIIT probably does not increase VO_2max_ compared with MICT (Table 5).

### 3.6 Quality of life

QoL was assessed in four systematic reviews. Two of the studies showed that an intervention adding HIIT versus OT to routine cancer treatment as prehabilitation^45^ or rehabilitation^51^ improved QoL, while the remaining two studies showed controversial results adding HIIT as prehabilitation and rehabilitation.^49^, ^50^ On the one hand, Mugele et al. found no statistically significant differences in two of their primary studies, while one did find differences in some 36‐ item short form survey subscales in favor of HIIT.^49^ On the other hand, Blackwell et al. showed an increase in QoL when analyzing a HIIT versus OT, however, when comparing HIIT versus MICT, they observed that mental health subscales improved with HIIT, while physical health subscales improved for MICT.^50^

### 3.7 Adherence and/or adverse events

Five systematic reviews evaluated adherence to the HIIT‐based intervention.^45^, ^46^, ^48^, ^49^, ^51^ In three of the studies, adherence was high, ranging from 71% to 100% during prehabilitation HIIT^45^ or prehabilitation and rehabilitation.^48^, ^49^ The remaining two studies had slightly lower adherence, ranging from 54 to 97%,^46^, ^51^ during prehabilitation or rehabilitation, respectively. Tsuji et al. observed that adherence was lower in cancer survivors, ranging from 57% to 75%, compared with adherence during active treatment, ranging from 57% to 97%.^51^

In relation to the adverse events of the HIIT intervention, four systematic reviews evaluated this outcome.^45^, ^46^, ^48^, ^51^ In most of the studies there were no adverse effects of any kind. Only a few primary studies presented mild adverse effects, mainly related to discomfort during exercise, acute post‐exercise pain, nausea, or blood pressure alterations.

## 4 DISCUSSION

The aim of this umbrella review and MMA was to analyze the effectiveness, safety, and feasibility of HIIT added to the first‐choice cancer treatment on CRF, QoL, adherence level, and its safety in patients with cancer or cancer survivors. The results showed a statistically significant increase in VO_2max_ when adding HIIT compared with OT, but no statistically significant difference when compared with adding MICT. Similarly, on QoL, first‐choice treatment plus HIIT compared with OT did show differences, but compared with MICT, its benefits were unclear. Adherence to the HIIT intervention was adequate and, in addition, there were very few and mild adverse events.

The clinical relevance of these CRF findings lies in the fact that cancer is one of the leading causes of mortality and, in addition, cancer survivors have a significant risk of death from cardiovascular diseases. CRF is considered an important predictor for survival in patients with cancer or cancer survivors, although the association between CRF and mortality risk may vary depending on the type of cancer, due to cancer‐specific biological mechanisms. Schmid and Leitzmann found a significantly decreased risk of mortality in patients with cancer with high versus low CRF (relative risk [RR] = 0.55; 95% CI 0.47 to 0.65) and moderate versus low CRF (RR = 0.80; 95% CI 0.67 to 0.97).^52^

It appears that moderate to high intensity resistance exercise has better results than light intensity in terms of its effectiveness on tumor factors.^53^ However, the results of this study showed that no such differences were found between moderate and high intensity training, with moderate evidence. An important factor to take into account is that the intervention used in the primary studies of the reviews was mostly aerobic HIIT. Only the systematic reviews by Palma et al. and Tsuji et al. included resistance exercise in combination with HIIT.^45^, ^51^

HIIT has shown a 10–13% increase in CRF or improved QoL in cancer survivors after chemotherapy treatment.^54^ Future research in the oncology population could evaluate whether specific type of HIIT is superior, in terms of CRF, fatigue or QoL, or whether the combination of both could enhance treatment outcomes. Structured exercise models employing HIIT have been widely studied in the literature and have showed to have positive results in different pathologies, such as cardiometabolic disease, cardiovascular disease, or diabetes compared with MICT.^55^, ^56^, ^57^ However, some difficulties that must be considered may arise when implementing exercise in patients with cancer or cancer survivors. Age or disease treatments may affect processes related to oxygen supply. It could result in exercise intolerance or limited exercise capacity.^58^ Structured aerobic exercise has been proposed to try to mitigate this exercise intolerance.^58^

The antitumor mechanism of exercise or its effect on patients with cancer is not yet fully understood, partly due to the observational design of most of the studies that address it.^59^ It seems that the antitumor effect derives from the influence of exercise on regulatory mechanisms of the tumor microenvironment, such as angiogenesis or immune regulation, as well as from increased blood perfusion and reduced tumor hypoxia.^60^ Some findings in the current scientific literature suggest promising results added to the first‐choice treatment. At the biological level, tumor cells present cell's metabolism alteration favoring cancer progression.^61^ Due to the energy expenditure involved, it seems that exercise influences intratumoral metabolism, biological mechanisms and some of the cellular processes associated with cancer.^53^ Besides that, the change in VO_2max_ has been considered the variable to be analyzed to determine the effectiveness of exercise‐based interventions, whether aerobic or endurance.^62^ VO_2max_ could influence tumor biology, since the tumor environment is usually hypoxic, and such hypoxia could reduce the response to treatment and, therefore, the prognosis of the disease.^63^ Thus, exercise generates adaptations at the systemic level related to hypoxia, vascularization or reduction of oxidative stress. Those adaptations may influence the tumor and even the response to adjuvant treatment.^64^ Tumor and host‐related characteristics could be determinant in the response of patients with cancer to exercise therapy.^59^ Further study of this aspect would allow us to understand the mechanisms of action and to propose individualized exercise models. HIIT, as an exercise model, would comply with these physiological underpinnings and therefore act in the same way on the cancerous process.

Given that our results did not show that the intensity and intervallic or continuous pattern could have a potential role in the effectiveness of exercise in CRF and QoL, other aspects to be taken into account should be evaluated to determine which type of exercise would be more favorable in patients with cancer or cancer survivors. The evaluation of adverse effects and adherence to the HIIT intervention allows us to assess its safety and feasibility. One of the main barriers described by patients to physical exercise is often lack of time. In relation to this, HIIT allows similar benefits to MICT, but involves less time.^65^ In addition to being more time‐effective, it can have cost benefits by decreasing the treatment time of each patient. Another common problem is a lack of motivation to exercise. HIIT has shown higher rates of perceived enjoyment than continuous exercise.^66^ It has been shown to increase enjoyment in sedentary subjects over a 6‐week training, whereas enjoyment with MICT was maintained or decreased.^67^ Therefore, implementing exercise patterns that are not too time‐consuming and enjoyable would increase exercise adherence. Since patients with cancer or cancer survivors are prone to physical inactivity, it is critical to ensure adherence to maintain exercise long enough to achieve benefits in CRF or QoL. Safety is also an important factor of concern to both clinicians and patients themselves.^65^ It has been shown to be a safe training since it has little or no adverse effects.

However, it is still necessary to know in depth the specific biological mechanisms that HIIT could produce compared with MICT in patients with cancer or cancer survivors. This will make it possible to find out whether, in addition to controlling the adverse effects derived from the disease, HIIT could enhance the effect of neoadjuvant cancer treatment. If true, furthermore, determining the host and tumor factors that could modulate the response to exercise would allow the evaluation of patients who are candidates for a type of exercise and the use of interventions based on tailored exercise models basis.

**FIGURE 5:**
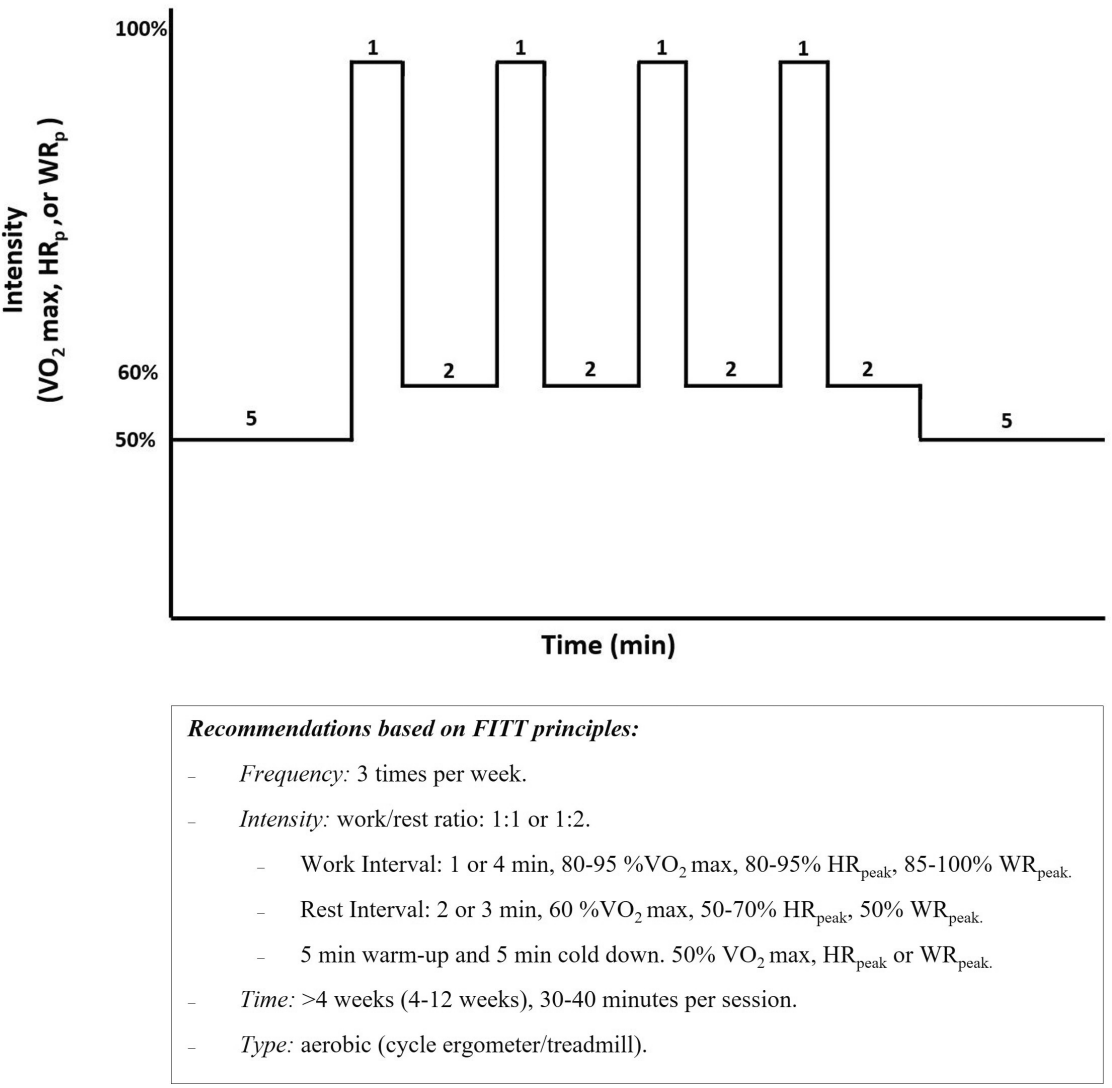
Recommendations for implementation of high‐intensity interval training in cancer patients based on FITT principles. HR_peak_, peak heart rate; VO_2max_, maximal oxygen uptake; WR_peak_, Work rate peak

Regarding the parameters of exercise application, the analysis of each of the different HIIT protocols in the primary studies would allow us to offer some recommendations based on the most common use of this type of exercise, which may be useful for application in the clinical setting (Figure 5). This synthesis of the current literature is intended to contribute positively to the development of future research on the effectiveness, safety, and feasibility of HIIT in patients with cancer or cancer survivors. In addition, it may provide a starting point for the development of future experimental studies that specifically evaluate which application parameters are most effective in different cancer populations.

### 4.1 Limitations

This umbrella review and MMA has some limitations. First, the small number of studies, as well as their low methodological quality and high risk of bias could have influenced the results. In addition, due to the low number of studies, it was not possible to quantitatively synthesize the evidence regarding QoL, adherence to treatment and adverse events. Second, despite the absence of heterogeneity in the MMA, the studies showed variability in terms of the type of cancer included and the HIIT protocol used. This has not made it possible to analyze each type of cancer and/or exercise in isolation. Although all reviews studied aerobic HIIT, it was not possible to analyze the type of exercise in terms of intensity, frequency, and time. Third, regarding the methodological design, the analysis of the influence of duplicity carried out reveals some problems derived from the statistical pooling of data extracted from systematic reviews. In the two MMA performed in our study, this problem does not affect the presence or absence of statistically significant differences and the variation in effect size is small. It is possible that this is due to the low number of primary studies included. This factor should be taken into account in the interpretation of the findings. In addition, one of the main limitations of the studies currently being carried out in patients with cancer or cancer survivors derives from the methodological design.^59^ Observational studies stand out, so the results derived from them should be interpreted with caution. Preclinical and clinical phase I, II, and III studies must be carried out. To this end, we have showed that exercise intervention, including high intensity design, are safe to implement in people with oncological pathology.

### 4.2 Perspective

The findings show the therapeutic potential of HIIT addiction in the treatment of patients with cancer or cancer survivors to improve CRF or QoL. These results on the efficacy of HIIT were similar to those shown in previous systematic reviews. However, the methodological design of the umbrella review and MMA allowed us to assess other issues in addition to the effectiveness of HIIT. We were able to analyze the quality of the available reviews, as well as to detect issues that have not yet been addressed in existing systematic reviews regarding HIIT in patients with cancer or cancer survivors. The possible influence of different types of cancer or the first‐choice cancer treatment on the effectiveness of HIIT has not yet been studied. Neither has the type and parameters of exercise used been analyzed in depth, with the aim of being able to provide recommendations on the most effective HIIT mode of use in terms of FITT principles. It would also be interesting to find possible predictors of HIIT efficacy in patients with cancer or cancer survivors. This highlights the need for future research questions including sub‐analyses and complementary analyses. Future research could address, in addition to the effectiveness of HIIT in general, its effectiveness according to different factors related to the type of patient, cancer and treatment or the exercise prescription itself.

We hope that the findings and concerns of this umbrella review will establish a basis and provide a proposal for improvement for future systematic reviews. Future research groups will be able to know which questions have already been studied and have a conclusive answer and focus their research on those questions that have not yet been clarified. This will avoid wasting financial and human resources on repeating reviews similar to the current ones.

## 5 CONCLUSION

There is moderate evidence that adding HIIT to the first‐choice cancer treatment improves CRF in patients with cancer or cancer survivors compared with adding OT, but no significant difference was found compared to MICT. Positive but controversial results were also found in terms of QoL and, exercise adherence to the HIIT intervention was adequate. Most studies reported no adverse events, and in the few cases in which there were, they were mild.

Therefore, although current evidence shows benefits of adding HIIT to routine treatment in patients with cancer or cancer survivors, further research is needed to ensure the effectiveness, safety, and feasibility of its use.

## AUTHOR CONTRIBUTIONS

FCM involved in conceptualization. AHG, CVR, LSM, and JCG involved in data curation. CVR involved in formal analysis and software. FCM, JCG, JC, and MBD involved in investigation. FCM and CVR involved in methodology and resources. JCG and JC involved in project administration. FCM, JCG, and LSM involved in supervision. LSM, JC, and MBD involved in visualization. AHG and CVR involved in roles/writing—original draft. AHG, FCM, CVR, LSM, JC, MBD, and JCG involved in writing—review and editing.

## FUNDING INFORMATION

None.

## CONFLICT OF INTEREST

None.

## Data Availability

Data sharing is not applicable to this article as no new data were created or analyzed in this study.

## APPENDIX 1 Database search strategies

***PubMed:***

((((((Cancer) OR (Malignant neoplasm)) OR (Malign neoplasm)) OR (Malignant tumor)) OR (Malign tumor)) OR (Oncology)) AND (((((((High‐intensity interval training[MeSH Terms]) OR (High intensity interval training)) OR (High‐intensity interval exercise)) OR (High‐intensity intermittent training)) OR (High‐intensity intermittent exercise)) OR (HIIT))) Filters: Meta‐Analysis, Systematic Review.

***EMBASE:***

(**“high intensity interval training”**/exp OR **“high intensity interval training”**) AND (**“malignant neoplasm”**/exp OR **“malignant neoplasm”**) AND ([cochrane review]/lim OR [systematic review]/lim OR [meta analysis]/lim) AND [1966–2021]/py.

(**“high intensity interval training”**/exp OR **“high intensity interval training”**) AND (**“cancer therapy”**/exp OR **“cancer therapy”**) AND ([cochrane review]/lim OR [systematic review]/lim OR [meta analysis]/lim) AND [1967–2021]/py.

***Cochrane Database of Systematic Reviews:***

#1 “High intensity interval training” OR “HIIT” in Cochrane Reviews

#2 MeSH descriptor: [High‐Intensity Interval Training] explode all trees

#3 “Cancer” in Cochrane Reviews

#4 MeSH descriptor: [Neoplasms] explode all trees

#5 #1 OR #2 in Cochrane Reviews

#6 #3 OR #4 in Cochrane Reviews

#7 #5 AND #6 in Cochrane Reviews

***CINAHL:***

(hiit or hit or high intensity interval training or high intensity training) AND (cancer or tumor or neoplasm) AND (systematic review or meta‐analysis)

***Scopus:***

TITLE‐ABS‐KEY (“high intensity interval training” OR “HIIT”) AND TITLE‐ABS‐KEY(“cancer”) AND TITLE‐ABS‐KEY (“systematic review” OR “meta‐analysis”).

***SPORTDiscus:***

(hiit or hit or high intensity interval training or high intensity training) AND (cancer or cancer patients or neoplasm or tumor) AND (systematic review or meta‐analysis)

***Web of Science:***

TS = (“High intensity interval training” OR “HIIT”) AND TS = (“cancer” OR “neoplasm”) AND TS = (“systematic review” OR “meta‐analysis”).

***Google Scholar:***

Field: “with all of the words;” filter: “in the title of the article.”

“high‐intensity interval training” AND cancer AND (systematic review OR meta‐analysis).

“high‐intensity interval exercise” AND cancer AND (systematic review OR meta‐analysis).

## APPENDIX 3 Overlapping of primary studies within systematic reviews with or without meta‐analysis

**Table:**

		Systematic reviews	Palma et al., 2021	Smyth et al., 2021	Tsuji et al., 2021	Maginador et al., 2020	Wallen et al., 2020	Mugele et al., 2019	Blackwell et al., 2018	Duplicates
N°	Primary Studies									
1	Blackwell et al., 2020			●						1
2	Minnella et al., 2020			●						1
3	Lee et al., 2020				●					1
4	Mijwel et al., 2020				●					1
5	Bhatia and Kayser, 2019		●	●						2
6	Egegaard et al., 2019		●				●			2
7	Mijwel et al., 2019		●							1
8	Alizadeh et al., 2019a				●					1
9	Alizadeh et al., 2019b				●					1
10	Lee et al., 2019a				●					1
11	Lee et al., 2019b				●	●	●			3
12	Northey et al., 2018				●		●			2
13	Mijwel et al., 2018a				●					1
14	Mijwel et al., 2018b				●					1
15	Mijwel et al., 2018c				●					1
16	Schulz et al., 2018				●					1
17	Adams et al., 2018							●		1
18	Devin et al., 2018							●		1
19	Ma, 2018					●				1
20	Banerjee et al., 2017		●	●			●			3
21	Karenovics et al., 2017		●	●				●		3
22	Licker et al., 2016			●			●	●	●	4
23	Sebio Garcia et al., 2017			●						1
24	Adams et al., 2017						●	●		2
25	Brunet et al., 2017							●		1
26	Dunne et al., 2016		●	●			●		●	4
27	Dolan et al., 2016				●		●	●	●	4
28	Schmitt et al., 2016						●	●	●	3
29	Devin et al., 2016						●	●	●	3
30	Toohey et al., 2016							●		1
31	West et al., 2015		●				●	●		3
32	Hwang et al., 2012						●	●	●	3
33	Adamsen et al., 2009		●							1

The left column shows each of the primary studies (randomized controlled trials or controlled clinical trials) included in the reviews. The following columns show the systematic reviews included in the umbrella review, indicating with a dot the primary studies that included each of them.

## APPENDIX 5 Analysis of duplicity for cardiorespiratory fitness for: (A) High‐intensity interval training (HIIT) training against other treatments (OT), (B) HIIT against moderate‐intensity continuous training (MICT)

This forest plot summarizes the results of included studies (standardized mean differences [SMDs], and weight). The small boxes with the squares represent the point estimate of the effect size and sample size. The lines on either side of the box represent a 95% confidence interval (CI).
